# A pediatric case of hypomagnesemia 1 (HOMG1) caused by novel compound heterozygous mutations in *TRPM6*

**DOI:** 10.1038/s41439-019-0043-0

**Published:** 2019-03-06

**Authors:** Takeshi Goda, Hiroshi Komatsu, Kandai Nozu, Hisakazu Nakajima

**Affiliations:** 1 Department of Pediatrics, National Hospital Organization Maizuru Medical Center, Kyoto, Japan; 2 Department of Pediatrics, Kobe University Graduate School of Medicine, Hyogo, Japan; 3 Department of Pediatrics, North Medical Center Kyoto Prefectural University of Medicine, Kyoto, Japan

**Keywords:** Acid, base, fluid, electrolyte disorders, Genetics research, Endocrine system and metabolic diseases

## Abstract

Hypomagnesemia 1 (HOMG1) is an extremely rare disease with autosomal recessive inheritance that is caused by mutations in the transient receptor potential melastatin 6 gene (*TRPM6*). Here, we describe a pediatric HOMG1 case with novel compound heterozygous mutations of *TRPM6* (c.1483 C > T [p.Gln495*] and c.2715del [p.Trp905*]) in a 2-month-old boy who developed refractory seizures due to hypomagnesemia with secondary hypocalcemia.

## Case report

Hypomagnesemia 1 (HOMG1: OMIM # 602014) is a rare autosomal recessive disease characterized by a high reduction of serum magnesium levels and is often accompanied by secondary hypocalcemia. In general, HOMG1 patients develop generalized convulsions, tetany, or neuromuscular excitability in early infancy^1^. The first case report of an HOMG1 patient was described by Paunier et al.^2^. Schlingmann et al. reported that mutations in the transient receptor potential melastatin 6 gene (*TRPM6*), which is located on chromosome 9q22, are responsible for HOMG1^3^. The TRPM6 protein has a crucial role in magnesium metabolism in human and mammals. Magnesium absorption predominantly occurs in the small intestine. The TRPM6 protein is an epithelial magnesium channel expressed along the brush border membrane of the small intestine. Intestinal absorption of magnesium occurs according to paracellular simple diffusion via concentration gradient and active transcellular uptake through the magnesium transporter of the TRPM6 protein at a low concentration of magnesium. Moreover, reabsorption of magnesium occurs in the kidney. The TRPM6 protein also plays an essential role in the apical influx of magnesium ion in the distal convoluted tubule cells^4^. Impaired channel activity of the TRPM6 protein caused by *TRPM6* mutations induces hypomagnesemia in infancy. Several mutations have been reported in families with HOMG1. Here, we describe a pediatric case of HOMG1 with novel compound heterozygous mutations.

**Fig. 1:**
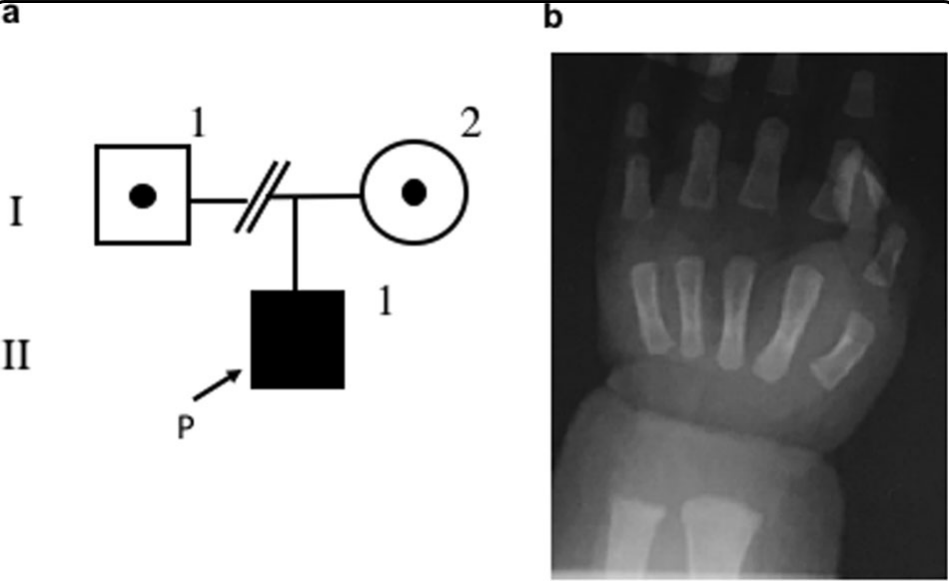
Pedigree chart and roentgenogram. **a** The family pedigree; the arrow indicates the proband (P). **b** Radiograph showing a “cupping” finding in the distal ulnar

A 2-month-old boy suffering from refractory seizures was referred to our hospital. He was born to non-consanguineous Japanese parents at a gestational age of 39 weeks after an uneventful pregnancy and delivery. Birth weight and birth height were 3342 g (+1.1 SD) and 50.5 cm (+0.9 SD), respectively. There were neither epileptic disorders nor electrolyte disorders in his family history. On admission, we found hypocalcemia (serum ionic calcium, 3.16 mg/dL; normal range, 4.60–5.17 mg/dL) and hypomagnesemia (serum magnesium 0.6 mg/dL; normal range, 1.8–2.4 mg/dL). Initial data of fractional excretion of magnesium (FEMg) was 0.09% (normal range, 3–5% for normomagnesemic individuals). Serum inorganic phosphate was 6.9 mg/dL (normal range, 4.5–6.2 mg/dL), and alkaline phosphatase was 1290 U/L (normal range, 480–1620 U/L). Although serum intact parathyroid hormone (PTH) was 80 pg/mL (normal range, 10–65 pg/ml) and 1,25-dihydroxyvitamin D_3_ was 85 pg/mL (normal range, 20–70), serum 25-hydroxyvitamin D_3_ was insufficient (4.1 ng/mL; normal range, 10–30 ng/mL). Brain MRI and cerebrospinal fluid examination revealed no abnormalities. Replacement therapy with intravenous infusion of calcium gluconate and magnesium sulfate was successfully performed to suppress the seizures. Calcium infusion therapy was discontinued when normal calcium levels were achieved. FEMg was elevated up to 4.93% during magnesium intravenous infusion, and serum magnesium was 1.6 mg/dL. He was discharged after switching from intravenous magnesium replacement to oral magnesium administration (magnesium oxide, 90 mg/kg/day). The Ellsworth–Howard test disclosed a normal response of urinary cAMP excretion to PTH stimulation (urinary cAMP level: before PTH injection, 570 pmol/mL; 1 h after PTH injection, 99,000 pmol/L). These results do not suggest the presence of pseudohypoparathyroidism. FEMg values ranged from 0.16 to 1.88% (median, 0.76%; normal range, 3–5% for normomagnesemic individuals). The urinary calcium/creatinine ratio ranged from 0.01 to 0.23 (median, 0.1; normal range, <0.81). A roentgenogram showed a cupping lesion in the distal ulnar (Fig. 1). Since oral alfacalcidol (a derivative of vitamin D_3_) treatment was not able to maintain serum ionic calcium concentration within the normal range, we discontinued it. At an age of 1 year, only oral magnesium treatment (magnesium oxide, 45–90 mg/kg/day) was successful in maintaining serum 25-hydroxyvitamin D_3_ levels within the normal range without vitamin D_3_ supplementation. We concluded that nutritional vitamin D_3_ deficiency was not the primary reason for hypocalcemia. However, hypomagnesemia recurred with reduction of oral magnesium administration (magnesium oxide, 20 mg/kg/day). Serum ionic calcium levels varied between 4.41 and 5.24 mg/dL, and magnesium levels were maintained between 1.3 and 1.7 mg/dL with treatment. At an age of 1 year and 8 months, he showed age-appropriate physical development and achieved developmental milestones. There were no adverse events of magnesium administration.

**Fig. 2:**
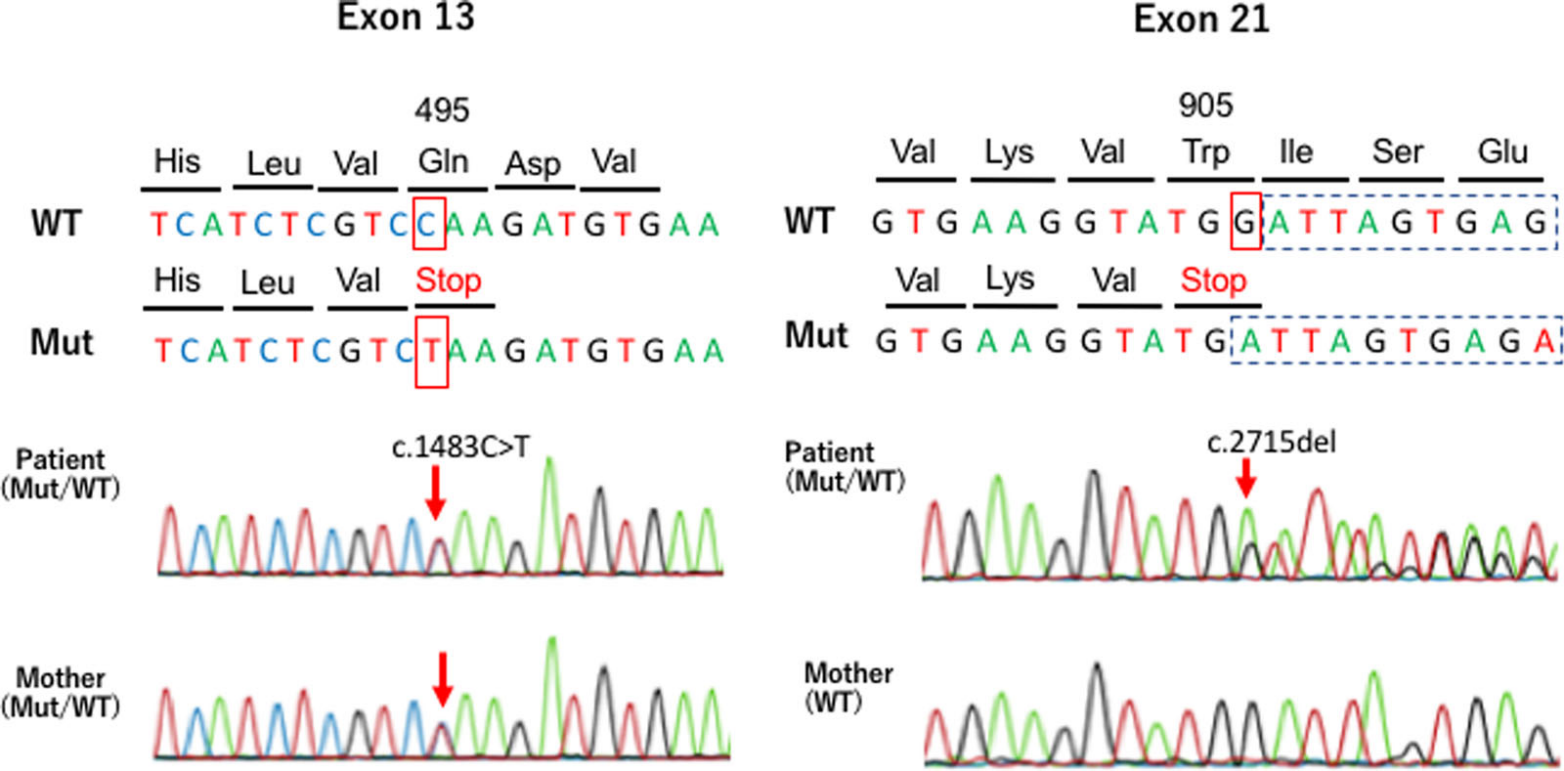
*TRMP6* mutations. The partial sequencing chromatograms demonstrate two novel mutations, c.1483 C>T [p.Gln495*] and c.2715del [p.Trp905*], in the *TRPM6* gene. The red arrow and red box represent the positions of the nucleotide mutations in exon 13 and exon 21, respectively. WT wild type, Mut mutation, Stop stop codon (termination codon)

We performed molecular genetic analysis on the proband and his mother after obtaining written informed consent from his mother. Genomic DNA was extracted from peripheral blood samples. The father’s blood sample was not taken because we could not contact the divorced father. Inherited hypomagnesemia-responsible genes including *TRPM6, CLCNKB, BSND, SLC12A3, CASR, KCNJ10, CLDN16, CLDN19, FXYD2, EGF, KCNA1, CNNM2*, and *HNF1B* were screened by next-generation sequencing analysis with targeting sequencing^5,6^. Finally, Sanger sequencing confirmed compound heterozygous mutations (NM_017662.5 (TRPM6_v001): c.1483C>T [p.Gln495*] and NM_017662.5 (TRPM6_v001): c.2715del [p.Trp905*]) in *TRPM6* (Fig. 2). p.Gln495* was also detected in his mother’s genome. These two novel mutations have not been documented in several open databases, including ClinVar (https://www.ncbi.nlm.nih.gov/clinvar/), dbSNP (https://www.ncbi.nlm.nih.gov/projects/SNP/), ExAc (http://exac.broadinstitute.org/about), and the Human Gene Mutation Database (http://www.hgmd.cf.ac.uk/ac/index.php). We used the GenBank transcript ID (NM_017662.5) as a reference to examine the pathological significance of the mutations.

We have described a pediatric case with novel compound heterozygous mutations of *TRPM6*, two of which also introduced a stop codon, namely the codon TGA/TAA. The mutations in the present case are predicted to cause premature termination of *TRPM6* coding. Theoretically, the mutated TRPM6 proteins in the present case are associated with loss of biological channel activity. *TRPM6* comprises 39 exons and encodes a large protein of 2022 amino acids^7^. The TRPM6 protein consists of six transmembrane segments as well as N-terminal and C-terminal domains. The p.Gln495 position of the amino acid sequence in the TRPM6 protein is located in the intracellular N-terminal domain. The p.Gln495* mutation predicts a truncated version of the TRPM6 protein that does not contain part of the magnesium-ion gating channel. This prediction suggests a loss-of-function mutation. However, the p.Trp905* mutation due to a frameshift nucleotide change (c.2715del) is located in the cytoplasmic loop of the TRPM6 protein. The TRPM6 protein that results from the p.Trp905*mutation is also predicted to be non-functional. In addition, we found that the two mutations were novel mutations according to databases.

HOMG1 patients primarily exhibit impairment of intestinal absorption of magnesium^3^. Indeed, the initial value of FEMg on admission was extremely low in the current patient. FEMg increased to nearly the upper limit of the normal range when intravenous infusion of magnesium was continued. These findings suggested that renal magnesium reabsorption was also impaired in the patient. However, magnesium leakage in the kidney will lead to difficulty in achieving normal magnesium levels even if adequate supplemental treatment of magnesium is given orally in patients with HOMG1, as was observed in the present patient.

The index patient is a sporadic case of HOMG1 with a typical clinical presentation including refractory seizures. In general, magnesium greatly contributes to PTH release through cAMP elevation by adenylate cyclase activation in the presence of magnesium in the parathyroid gland^8^. Therefore, secondary hypocalcemia subsequently occurs in patients with HOMG1^9^. In that context, it is a clinical observation of great interest that serum intact PTH was elevated in the current case, although previous HOMG1 cases have shown inappropriately low levels of serum intact PTH despite hypocalcemia^10^. In the current case, the initial data of a low level of serum 25-hydroxyvitamin D_3_ and roentgenogram findings supported a diagnosis of calcipenic rickets induced by vitamin D_3_ deficiency. Moreover, we considered that serum calcium levels were dependent on magnesium administration in the current case. With respect to vitamin D_3_ metabolism, magnesium plays an essential role in the activation of 25-hydroxylase, which converts vitamin D3 to 25-hydroxyvitamin D3^11^. We speculated that magnesium deficiency could lead to secondary dysregulation of vitamin D_3_ metabolism.

## Data Availability

The relevant data from this Data Report are hosted at the Human Genome Variation Database at 10.6084/m9.figshare.hgv.2540
